# Death’s Doings in 1854

**Published:** 1855-02

**Authors:** 


### DEATH'S DOINGS IN 1854,

### IN NEW YORK, PHILADELPHIA, BALTIMORE AND BOSTON.

RATIO OF DEATHS TO PRESENT ESTIMATED POPULATION.

| | Deaths in 1854. | Estimated Population. | Ratio of deaths to Inhabitants. |
|---|---|---|---|
| New York . . . . . . . | 28,458 | 625,000 | 1 to 21.95 |
| Philadelphia . . . . . . . | 11,811 | 500,000 | 1 to 42.33 |
| Baltimore . . . . . . . . | 5,738 | 210,000 | 1 to 36.59 |
| Boston . . . . . . . . . | 4,418 | 100,000 | 1 to 36.21 |

The deaths from various prominent diseases in the four cities were as follows:

| | New York. | Philadelphia. | Balt. | Boston. |
|---|---|---|---|---|
| Consumption . . . . . . . | 2,990 | 1,389 | 931 | 769 |
| Convulsions . . . . . . . | 2,327 | 695 | 122 | 151 |
| Cholera . . . . . . . . | 2,459 | 601 | 2 | 255 |
| Cholera Infantum . . . . . | 1,455 | 633 | 393 | 81 |
| Cholera Morbus . . . . . | 281 | 126 | 129 | 26 |
| Diarrhœa . . . . . . . . | 1,106 | 211 | 46 | 54 |
| Dysentery . . . . . . . . | 827 | 443 | 253 | 147 |
| Scarlet Fever . . . . . . | 484 | 162 | 252 | 64 |
| Typhus and Typhoid . . . | 504 | 166 | 114 | 102 |
| Inflammation of Lungs . . . | 1,152 | 456 | 151 | 249 |
| Small Pox . . . . . . . . | 425 | 37 | 29 | 117 |
| Marasmus . . . . . . . . | 1,398 | 439 | 9 | 99 |
| Still Born . . . . . . . . | 1,540 | 529 | 345 | *— |
| Other Diseases . . . . . . | 11,510 | 5,924 | 2,962 | 2,304 |
| Total . . . . . . . . . . | 28,458 | 11,811 | 5,738 | 4,418 |
| Under 5 years . . . . . . | 15,593 | 5,874 | 2,887 | 1,987 |

The above figures show in round numbers that one person out of every twenty-two died in New York in 1854; one in every forty-two in Philadelphia; one in every thirty-seven in Boston.

But although they are figures and statistics, they do not prove that New York is a more sickly place than Philadelphia, for it is known that great numbers who die, are persons who have landed from foreign countries but a few days before their death; about a thousand foreigners land here every day.

3

STATISTICS OF OLD AGE.—The census of 1854 shows us that the oldest person then living in the United States was 140. This person was an Indian woman, residing in North Carolina. In the same State was an Indian aged 125; a negro woman, 111; two black slaves, 110 each; one mulatto male, 120; and several white males and females from 106 to 114. In the parish of Lafayette, La., was a female, black, aged 120. In several of the States there were found persons, white and black, aged from 110 to 115. There were in the United States, in 1850, 2,555 persons over 100 years. This shows that about one person in 9,000 will be likely to live to that age. There are now about 20,000 persons in the United States who were living when the Declaration of Independence was signed in 1776. They must necessarily be nearly 80 years old now, in order to have lived at that time. The French census of 1851, shows only 102 persons over 100 years old; though their total population was near 36,000,000. Old age is therefore attained among us much more frequently than in France.

